# Rapid Parallel Adaptation to Anthropogenic Heavy Metal Pollution

**DOI:** 10.1093/molbev/msab141

**Published:** 2021-05-05

**Authors:** Alexander S T Papadopulos, Andrew J Helmstetter, Owen G Osborne, Aaron A Comeault, Daniel P Wood, Edward A Straw, Laurence Mason, Michael F Fay, Joe Parker, Luke T Dunning, Andrew D Foote, Rhian J Smith, Jackie Lighten

**Affiliations:** 1 Molecular Ecology and Evolution Bangor, Environment Centre Wales, School of Natural Sciences, Bangor University, Bangor, United Kingdom; 2 Royal Botanic Gardens, Kew, Richmond, United Kingdom; 3 FRB-CESAB, Institut Bouisson Bertrand, Rue de l'École de Médecine, Montpellier, France; 4 Centre for Ecology, Evolution & Behaviour, Department of Biological Sciences, School for Life Sciences and the Environment, Royal Holloway University of London, Egham, United Kingdom; 5 School of Plant Biology, University of Western Australia, Crawley, WA, Australia; 6 National Biofilms Innovation Centre, Department of Biological Sciences, University of Southampton, Southampton, United Kingdom; 7 Department of Animal and Plant Sciences, University of Sheffield, Sheffield, United Kingdom; 8 Department of Natural History, Norwegian University of Science and Technology, NTNU University Museum, Trondheim, Norway; 9 Biosciences, University of Exeter, Exeter, United Kingdom

**Keywords:** parallel evolution, rapid evolution, heavy metal tolerance

## Abstract

The impact of human-mediated environmental change on the evolutionary trajectories of wild organisms is poorly understood. In particular, capacity of species to adapt rapidly (in hundreds of generations or less), reproducibly and predictably to extreme environmental change is unclear. *Silene uniflora* is predominantly a coastal species, but it has also colonized isolated, disused mines with phytotoxic, zinc-contaminated soils. To test whether rapid, parallel adaptation to anthropogenic pollution has taken place, we used reduced representation sequencing (ddRAD) to reconstruct the evolutionary history of geographically proximate mine and coastal population pairs and found largely independent colonization of mines from different coastal sites. Furthermore, our results show that parallel evolution of zinc tolerance has occurred without gene flow spreading adaptive alleles between mine populations. In genomic regions where signatures of selection were detected across multiple mine-coast pairs, we identified genes with functions linked to physiological differences between the putative ecotypes, although genetic differentiation at specific loci is only partially shared between mine populations. Our results are consistent with a complex, polygenic genetic architecture underpinning rapid adaptation. This shows that even under a scenario of strong selection and rapid adaptation, evolutionary responses to human activities (and other environmental challenges) may be idiosyncratic at the genetic level and, therefore, difficult to predict from genomic data.

## Introduction

Modification of the natural environment by humans has significant implications for biodiversity (Urban 2015; Ceballos et al. 2017; Helmstetter et al. 2020). Rapid habitat loss or environmental change can drive species to the brink of extinction, but also presents opportunities for adaptation and speciation (Johnson and Munshi-South 2017; Otto 2018; Ravinet et al. 2018; Szulkin et al. 2020). The ability of species to adapt to human-modified landscapes or activities is a key determinant of their viability in the Anthropocene (McNeilly and Bradshaw 1968; Antonovics and Bradshaw 1970; Wu and Bradshaw 1972; Macnair 1979; Hof et al. 2016; Reid et al. 2016; Bosse et al. 2017). Thus, a key question in evolutionary ecology is how repeatable and predictable adaptation is to human-altered habitats (Bay et al. 2018; Fitzpatrick et al. 2018; Therkildsen et al. 2019; Santangelo et al. 2020; Van Etten et al. 2020). To demonstrate that local adaptation has driven the evolution of distinct ecotypes, it is necessary to establish an association between fitness differences of populations and specific habitats. However, we can investigate genomic processes that might contribute to adaptation by examining the sequence-based signatures of selection associated with local adaptation. This can be accomplished even when reduced representation sequencing methods are used (Lowry et al. 2017). In such cases, examples of parallel colonization of habitats with novel selection pressures can support the hypothesis that specific genetic loci underpin local adaptation (Rundle et al. 2000; Jones et al. 2012; Ravinet et al. 2016; Nosil et al. 2018). A genomic approach can also discriminate between single or parallel origins of populations adapted to a specific habitat or selection pressure. Local gene flow between differentiated populations can obscure the true evolutionary relationships between them and lead to false inferences (Ravinet et al. 2016; James et al. 2021). Promising cases of rapid parallel adaptation do exist (e.g., Lescak et al. 2015; Marques et al. 2016; Alves et al. 2019), but few have ruled out the possibility of local gene flow creating the false impression of independent origins (Roda et al. 2013; James et al. 2021).

Instances where the same toxic chemicals and contaminants have been repeatedly introduced into the environment by humans in isolated locations can generate novel selection regimes that have the potential to promote parallel adaptation. Strong selection, caused by herbicides, pesticides, and heavy metals that contaminate soils and water bodies, is capable of producing extremely rapid adaptive responses (Antonovics and Bradshaw 1970; Wu and Bradshaw 1972; Macnair 1979; Hartley et al. 2006; Van Etten et al. 2020) and trade-offs (Xie and Klerks 2004), and may be particularly prone to triggering parallel responses as a result (MacPherson and Nuismer 2017). Indeed, there is evidence for rapid parallel adaptation from “ancient” standing genetic variation during adaptation to copper mine contamination in two populations of *Mimulus guttatus* (Wright et al. 2015; Lee and Coop 2017). In the Atlantic killifish, *Fundulus heteroclitus*, tolerance to marine pollution has evolved in four populations (Reid et al. 2016). The mutations underlying this resistance have evolved on at least two occasions, but migration between three of the four populations may have contributed to the spread of tolerance (Lee and Coop 2017). Convergent herbicide resistance across species is well documented, but there is more limited support for parallel origins within single species and the spread of resistance by gene flow has been harder to rule out (Kreiner et al. 2019; Van Etten et al. 2020).

Here, we present evidence for multiple recent and independent origins of heavy metal tolerance in the predominantly coastal plant *Silene uniflora* (sea campion). In Great Britain and Ireland, metal mining activities had largely ceased by the early 20th century, but the legacy of spoil heaps and soils contaminated with heavy metals forms a patchwork of highly localized and drastically altered environments across the landscape (Baker et al. 2010). Heavy metals, such as zinc, copper, cadmium, and lead, are highly toxic to plants, triggering oxidative stress, inhibition of growth and photosynthesis, and death (Küpper and Andresen 2016). As a result, many of these abandoned sites remain barren for hundreds of years after the mining itself has ceased (Baker 1974; Baker et al. 2010). Despite its largely linear coastal distribution, *S. uniflora* has managed to colonize a number of isolated inland mine spoils in various regions of Great Britain and Ireland—although only a small proportion of the >10,800 nonferrous mines in Great Britain harbor the species (Baker 1974, 1978; Baker and Dalby 1980; Gill 2018). A common feature of the mines that it inhabits is an elevated level of zinc. Experiments in the 1970s demonstrated that: 1) mine populations are more zinc tolerant than coastal populations; 2) mine plants exclude zinc from their shoots, and 3) zinc tolerance in each population is tightly correlated with the concentration of zinc found in local soils (Baker 1978). Furthermore, in a common garden experiment using zinc-enriched slag from a population in Morriston in Swansea, Baker (1974) demonstrated that the local mine plants grew and produced flowers normally, whereas coastal plants remained in a dwarfed state, developed chlorosis (yellowing due to lack of chlorophyll) and did not produce any flowers—even in slag that had been heavily diluted with sandy soil. The link between the zinc tolerance phenotype, local levels of environmental zinc, and reduced fitness of coastal plants in zinc-contaminated soils suggests that mine populations are locally adapted to their environment.

Given the generally coastal distribution and the isolated nature of the colonized mines, we hypothesized that the mine populations have independently adapted from the nearest coastal populations. Across four local mine-coast population pairs, we used growth experiments to determine whether mine plants are more tolerant to zinc toxicity than their nearest coastal counterparts. We combined a newly sequenced draft genome with reduced representation genotypes for 216 individuals, conducting population genetic analyses to establish the relationships between the populations and test the hypothesis that the mine populations had evolved independently multiple times, following dispersal from their physically closest coastal populations. Finally, we used these data to explore the extent to which evolution of the mine populations is controlled by a parallel/convergent molecular basis.

## Results and Discussion

### Anthropogenic Adaptation to Heavy Metal Contamination

**Fig. 1.**
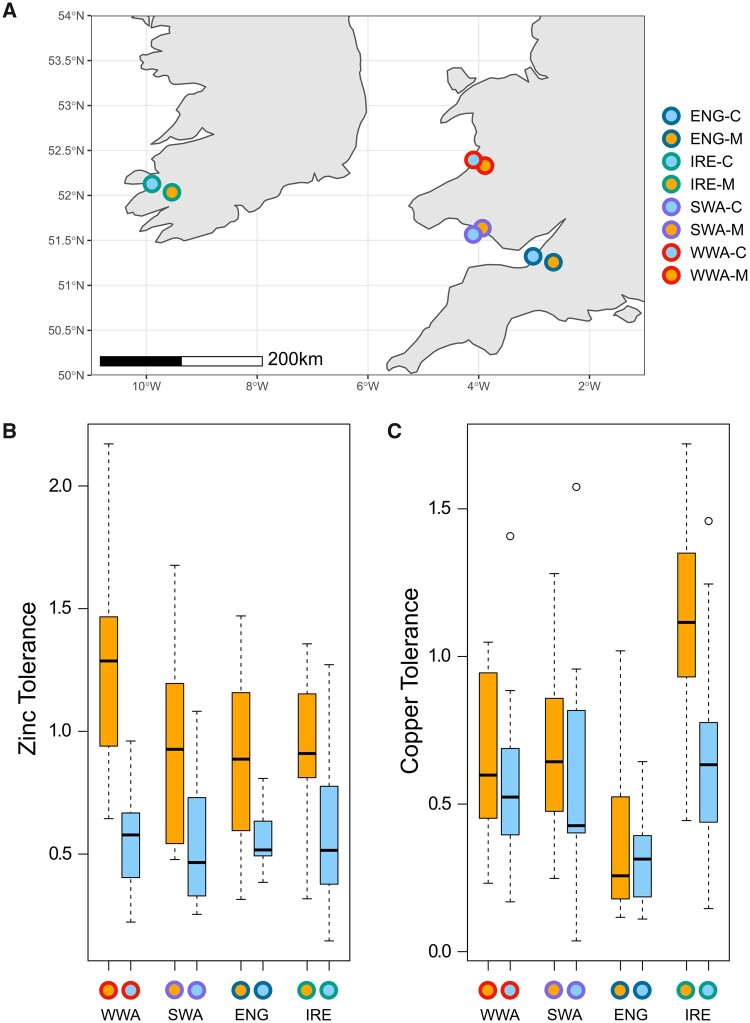
Differential heavy metal tolerance between local mine and coastal populations. (*A*) Map of population sampling locations. Fill colors denote habitat type (mine—orange, coastal—blue). Outline colors denote local populations (West Wales—WWA; South Wales—SWA; South-West England—ENG; South-West Ireland—IRE). The same color scheme is used throughout. (*B*) Zinc and (*C*) copper tolerance for each mine-coast pair (center line, median; box limits, upper and lower quartiles; whiskers, 1.5× interquartile range; points, outliers; zinc treatment left to right *n *=* *15/17/14/14/14/16/15/19; copper treatment left to right *n *=* *17/17/16/17/15/18/16/18). Local mine and coastal populations have significantly different zinc tolerance, but only the Irish pair have significantly different copper tolerance.

Populations of *S. uniflora* were sampled from four derelict mines and the nearest coastal population to each across Great Britain and Ireland (fig. 1*A*). Previous research has shown that the contaminated mine sites all have elevated toxic levels of zinc in the soil (2,410–48,075 ppm, supplementary table S1, Supplementary Material online) relative to typical coastal and inland sites (UK mean = 81.3 ppm; Ross et al. 2007). Lead levels were also high at all mine sites (>10,000 ppm, supplementary table S1, Supplementary Material online; UK mean = 52.6; Ross et al. 2007), but only the South Wales (SWA-M) and Irish (IRE-M) mines were heavily contaminated with copper (>10,000 ppm, supplementary table S1, Supplementary Material online; UK mean = 20.6; Ross et al. 2007). We used root elongation experiments with wild-collected seed to determine whether mine populations were more tolerant of zinc and copper than the most geographically proximate coastal population. In all cases, mine populations were significantly more zinc tolerant than the local coastal population (Welch’s *t*-test, two-sided, *P* < 0.005 for all four pairs; fig. 1*B*). Deep water culture experiments with cuttings from individuals grown in standard conditions also confirmed that plants from mine populations were more zinc tolerant than coastal populations: that is, root growth continued in mine plants at 600 µM ZnSO_4_, but not in coastal plants (see Materials and Methods). However, only the Irish mine population was significantly more copper tolerant than the respective local coastal population (Welch’s *t*-test, two-sided, *P* < 0.001, fig. 1*C*). The lack of clear copper tolerance in SWA-M may be due to the relatively high copper concentration used in the experiment, possibly beyond levels that can be tolerated by this population. It is notable that both mine and coastal populations from Wales were more copper tolerant than the English populations (fig. 1*C*), suggesting that SWA-M may be able to cope with high copper levels due to constitutive copper tolerance in Welsh *S. uniflora*. High intraspecific variation in copper tolerance has been observed in other species—even within a single mine (e.g., *Scopelophila cataractae*)—as has constitutive tolerance in non-mine specialists (e.g., *Ceratodon purpureus*; Boquete et al. 2021). Overall, these results corroborate earlier findings of zinc and copper tolerance in mine populations of *S. uniflora* (Baker 1978).

Although our experiments do not provide a direct measure of fitness in the wild, given the association between zinc tolerance, levels of zinc contamination in soil, vegetative growth, and flower production in *S. uniflora* (Baker 1974; 1978), our results indicate that all of the sampled mine populations are adapted to zinc contamination. Due to the strong selection that heavy metal toxicity exerts, tolerance can evolve in plants within as little as a single generation if there is sufficient genetic variation (Wu and Bradshaw 1972). Although limited mining activity existed at some of these sites as far back as the Bronze Age, the most intensive working took place between the 18th and 19th centuries (see Materials and Methods) and so it is likely that these anthropogenic mine habitats only became available for colonization once active excavation ceased at mining sites within the last 250 years (Baker 1974). Therefore, populations of zinc-tolerant *S. uniflora* studied here are likely to have evolved since the 18th century (i.e., <250 generations).

### Independent, Parallel Origins of the Mine Populations

In total, 216 individuals (*n* per population; WWA-M = 25, WWA-C = 28, SWA-M = 28, SWA-C = 27, ENG-M = 26, ENG-C = 27, IRE-M = 28, IRE-C = 27) were genotyped at 74,064 SNPs. On average “local” mine and coastal populations were 20.8 km apart (WWA = 16.1 km, SWA = 14.8 km, ENG = 25.6 km, IRE = 26.8 km). Genetic differentiation between populations was high (mean *F*_ST_ = 0.36; supplementary table S2, Supplementary Material online), reflecting the relatively poor dispersal capabilities and fragmented distribution of the species (Baker 1974; Runyeon and Prentice 1997). Differentiation was substantially higher between mine populations (mean *F*_ST_ = 0.45) than between coastal populations (mean *F*_ST_ = 0.25). Mine populations were also substantially differentiated from their local coastal population (mean *F*_ST_ = 0.36), suggestive of very limited gene flow between differentially adapted populations at the local level. In support of this, analysis of molecular variance (AMOVA; supplementary table S3, Supplementary Material online) shows that most of the variation is partitioned within and among individuals (∼65%), but a large proportion of variation was among populations which were grouped by either habitat (34%) or region (33%). Partitioning of genetic variation was low between habitats (1.5%) and fractionally larger between regions (2.0%), reflecting the very high differentiation between mines and greater degree of shared variation between local mine and coastal populations. Genetic diversity (π) was also significantly higher in the coastal populations versus the mine populations (0.065 and 0.044, respectively; Welch’s *t*-test, two-sided, *P* < 0.036, supplementary table S4, Supplementary Material online). Tajima’s *D* was slightly positive across all populations (mean = 0.24, supplementary table S4, Supplementary Material online), but not significantly different between the mine and coastal populations. As Tajima’s *D* is close to zero, the drop in diversity is unlikely to result from a population bottleneck, but this pattern matches expectations for multiple soft selective sweeps taking place across the genome (Pennings and Hermisson 2006)—as might be expected when colonizing a new environment in the face of a strong selection pressure with limited time for new adaptive mutations to evolve.

**Fig. 2.**
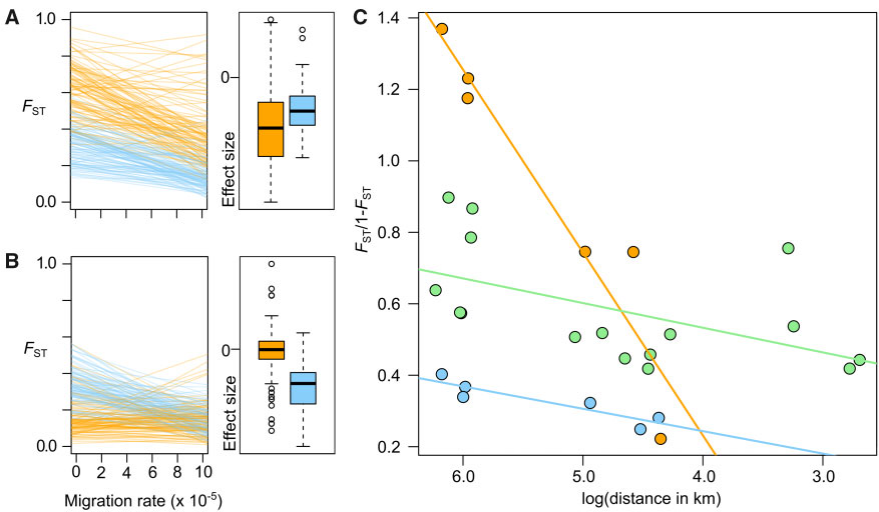
Isolation by distance (IBD) patterns arising from multiple or single origins of mine populations. (*A*) Under a simulated multiple independent origin model, the correlation between *F*_ST_ and migration between mine populations (orange) is steeper (i.e., IBD is stronger) and has a higher intercept than isolation by distance between coastal populations (blue). (*B*) In contrast, under a single origin model, the relationship between genetic differentiation and geography breaks down between mine populations—the slope is not significantly different from zero and the intercept is lower than between coastal populations (center line, median; box limits, upper and lower quartiles; whiskers, 1.5× interquartile range; points, outliers). (*C*) The observed IBD relationships in *Silene uniflora* conform to the patterns expected from multiple origins of the mine populations. IBD between mine and coastal populations in green.

In the context of recent colonization, relatively high differentiation and limited gene flow between populations, we predicted that different colonization scenarios would produce differing patterns of isolation by distance among mine versus coastal habitats (IBD; Wright 1943; James et al. 2021)—specifically that a scenario of independent origins of the mine populations would be distinguishable from a single origin. In a multiple origin scenario, IBD among mine populations should be accentuated relative to the pattern across coastal populations, whereas, in a single origin scenario, IBD among mine populations should be minimal. To test these predictions, we conducted forward-in-time simulations in SLiM v3 (Haller and Messer 2019) and estimated within-habitat IBD under “multiple-origin” and “single-origin” colonization scenarios (fig. 2*A* and *B*, see Materials and Methods). As expected, the strength of IBD was significantly higher among the mine populations than among the coastal populations for the multiple origin scenario (paired *t*-test, two-sided, *P* < 0.001; fig. 2*A*) and the reverse was true for the single origin scenario (paired *t*-test, two-sided, *P* < 0.001; fig. 2*B*). The observed IBD in the sampled populations (fig. 2*C*) closely matches the expectations for a parallel origin of mine populations, supporting the hypothesis that the mine habitat has been colonized independently.

**Fig. 3.**
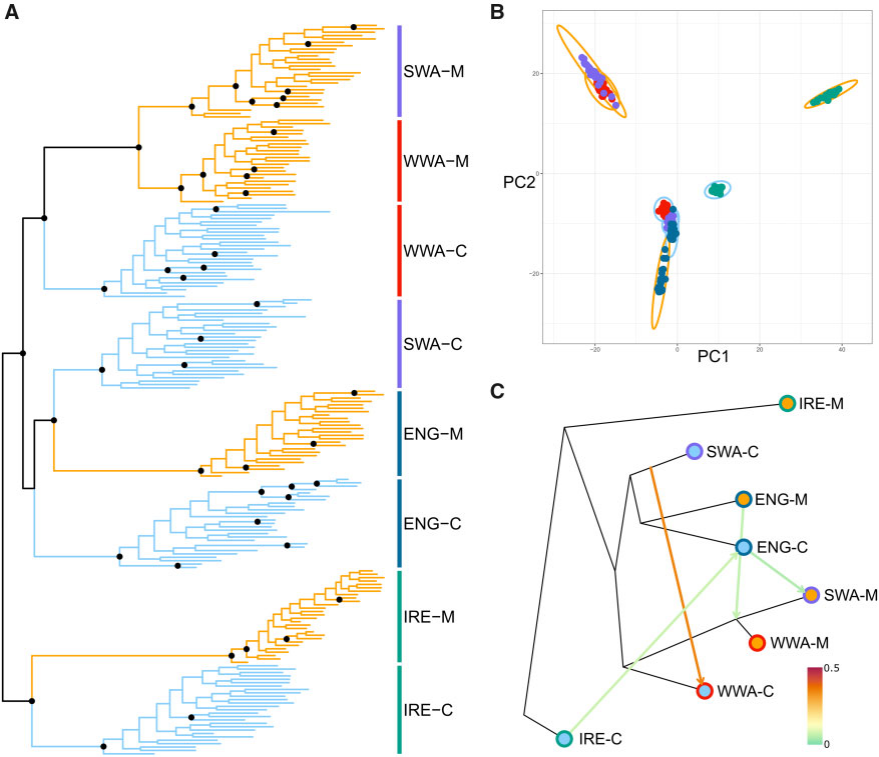
Evidence for three independent origins of zinc-tolerant populations in *S. uniflora.* (*A*) Phylogenetic reconstruction (mine populations in orange and coastal populations in blue). Nodes with greater than 90% bootstrap support are denoted by black circles. (*B*) PCA points to three, well-supported, independent origins of zinc-tolerant populations. Variance explained by PC1 = 12.3% and PC2 = 9.0%. Points are colored by region as figure 1. All points from a specific population are surrounded by a single ellipse which is colored by habitat type (mine—orange, coast—blue). (*C*) Treemix analysis with four migration edges. Points are colored as in figure. 1. The topology is almost identical to that produced by the SNPhylo analysis—the relationship of ENG-C and SWA-C to ENG-M is reversed. Color scale indicates migration edge weight. Only migration between coastal populations was supported by *f*4 statistics (see fig. 4).

Phylogenetic reconstruction of evolutionary relationships between the *S. uniflora* populations based on 7,037 linkage disequilibrium pruned SNPs (fig. 3*A*) and principal components analysis (PCA) of genetic structure from the full set of 74,064 genome aligned SNPs (fig. 3*B*), clearly indicate three independent origins of zinc-tolerant mine populations; one in Ireland, one in England, and one in Wales. The PCA highlights the much higher genetic similarity between coastal populations than between mine populations, which occupy extremely divergent areas of genotype space, suggesting that they may be on different evolutionary trajectories at the genetic level, despite adapting to similar selection pressures. The two Welsh mines are genetically similar (fig. 3*B* and supplementary fig. S1, Supplementary Material online) and although we cannot rule out independent origins from unsampled nontolerant populations, it is likely that transport of workers, machinery, or ore between Welsh mines dispersed zinc-tolerant plants between sites. In fact, records of mine ownership from 1758 C.E. indicate that human-mediated dispersal is possible between West Wales and Swansea and it was common practice to transport ore mined elsewhere to be refined in Swansea (Hughes 2000). There are at least 14 further records of *S. uniflora* growing on contaminated mine spoil in the UK and Ireland (pers. obs. and Baker 1974), so our discovery of three independent origins is likely to be a lower bound on the true number of independent origins for zinc-tolerant populations.

**Fig. 4.**
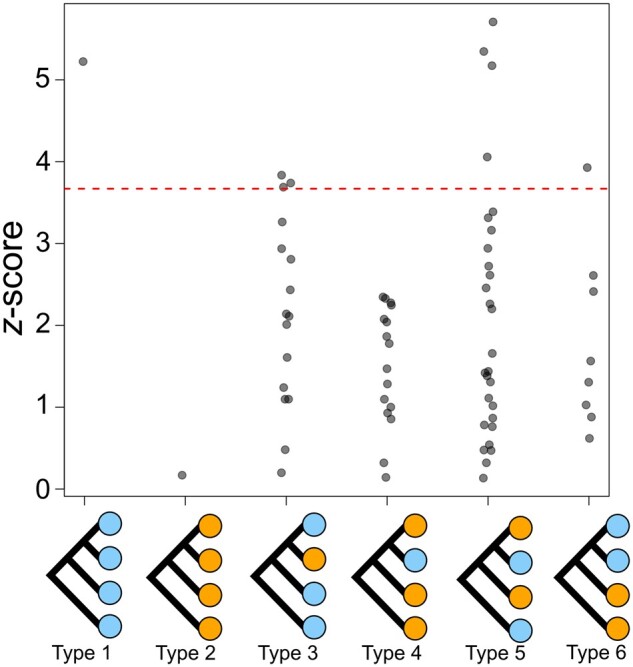
Evidence for admixture between coastal populations but not between mines. *z*-scores of *f*4 statistics for the six different permutations of four taxon trees (Types 1–6), with all of the different combinations of mine (orange) and coastal (blue) populations based on relationships in figure 3*A*. The red line denotes the *z*-score at which the *f*4 statistic is significantly different from zero at the 5% level after Dunn–Bonferroni correction for multiple tests (*z *=* *3.67). There is evidence of admixture in the *four coast tree* (Type 1; *z *=* *5.23) and *three coast: one mine trees* (Type 3; *z *>* *3.67 for three of the quartets), which is also reflected in the four Type 5 quartets with *z*-scores exceeding 3.67. On the other hand, the *four mine* (Type 2; *z *=* *0.17) and *three mine: one coast trees* (Type 4; *z *=* *0.14–2.35) demonstrate that there has not been introgression between mine sites.

Three origins of zinc-tolerant populations were further supported when modelling shared genetic drift among populations (Treemix analysis; fig. 3*C*). This analysis also provided evidence of migration between the Welsh coastal populations (WWA-C and SWA-C) and very weak migration between the Irish, English, and Welsh populations. To assess the significance of admixture in the evolution of the mine populations, we examined genetic relationships across all population quartets using the less-parameterized *f*4 statistics (fig. 4). The *f*4 statistic quantifies shared drift between pairs of populations in a four-taxon tree—significant deviation of the *f*4 statistic from zero for the tested topology demonstrates that the relationships are not perfectly described by a bifurcating tree. This is indicative of some shared drift between populations that conflicts with the topology, for example, due to admixture (Reich et al. 2009; Foote and Morin 2016; Peter 2016; Lipson 2020). The *f*4 statistic for the tree containing all four mine populations (type 2; fig. 4) indicates that there has been no admixture between mines (i.e., *f*4 does not deviate from zero; *f*4 = 1.31 × 10^−5^, s.d. = 7.75 × 10^−5^, *P* = 1.00), whereas *f*4 for the coastal population quartet (type 1; fig. 4) demonstrates that admixture between coastal populations has taken place (i.e., *f*4 is significantly different from zero; *f*4 = −3.95 × 10^−4^, s.d. = 7.57 × 10^−5^, *P* = 3.68 × 10^−5^). Comparisons of quartets with three mine populations and one coastal population (type 4; fig. 4) provide an additional test of the independent origins of the mine populations, in each case demonstrating that there was no correlated drift between the mine outgroups and the mine-coast pair of more closely related populations. On the other hand, the *three coastal: one mine* comparisons (type 3; fig. 4) provide further confirmation of gene flow from coastal outgroups into more closely related mine-coast pairs in three quartets and support the significance of migration edges between SWA-C and WWA-C, and IRE-C and ENG-C. Overall, our results provide firm support for recent parallel evolution of mine populations, with migration restricted to coastal sites.

### Evidence for Molecular Convergence/Parallelism

**Fig. 5.**
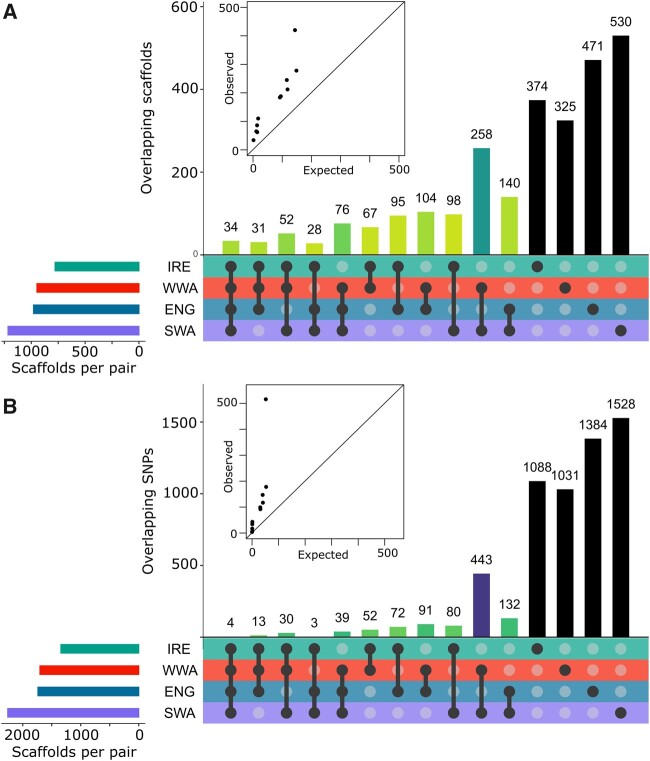
Molecular convergence and divergence across regional mine-coast pairs. Upset plots of the shared (*A*) outlier scaffolds and (*B*) individual SNPs across the four regional mine-coast pairs. Filled points below bars denote which regional sets are intersected for each bar (e.g., the leftmost bar in each plot represents the set including all four mine-coast comparisons). Inset scatterplots show observed overlap (*y*-axis) versus expected overlap (*x*-axis) across combinations of regional sets, with line at 1:1. Black bars denote outliers found in a single geographic region. The remaining bars are colored by super exact test *P* value (all < 0.001) with darker green denoting smaller *P* values and purple denoting extremely small values (<10^−150^).

To investigate the genetic basis of mine-coast differentiation and degree of molecular convergence in adaptation, we conducted pairwise *F*_ST_-based genome scans for each mine-coast pair and identified outlier loci potentially under divergent selection. Due to the relatively sparse sampling of our ddRAD data set and the highly fragmented draft genome (supplementary table S5, Supplementary Material online; N50 = 4,660 bp, length = 0.77 Gb), we designated genomic scaffolds containing at least one outlier SNP as an outlier scaffold for each comparison (the number of outlier SNPs was not significantly associated with scaffold length; Tukey’s test; supplementary fig. S2, Supplementary Material online). Across the local mine-coast pairs, the number of outlier scaffolds ranged from 779 to 1,216 and the number of outlier SNPs varied from 1,346 to 2,261—the degree of overlap between all sets of outlier scaffolds (fig. 5*A*) and SNPs (fig. 5*B*) was significantly higher than expected by chance as assessed by Super Exact Test (an extension of Fisher’s Exact Test for multiple sets; Wang et al. 2015). In total, 34 scaffolds were identified as outliers across all pairwise comparisons, whereas 187 and 756 outlier scaffolds were found across the sets of three and two comparisons, respectively (fig. 5*A*). There was substantially less overlap at the level of SNPs (fig. 5*B*), with four shared across all four sets, 85 shared by three sets and 870 shared by two sets. This pattern suggests a highly polygenic basis to mine-coast differentiation, with a substantial proportion of shared targets of selection found in three or fewer pairs. However, we are unable to rule out the possibility that the shared scaffolds are physically close to each other in the genome, although linkage disequilibrium between the scaffolds is low (mean *r*^2^ = 0.021).

It is currently unclear whether the adaptive variation that underpins tolerance and colonization of the mine habitat has arisen through new independent mutations in each population (as in *Fundulus heteroclitus*; Reid et al. 2016), has been drawn from standing variation (as in *Mimulus guttatus*; Lee and Coop 2017), or has been obtained through adaptive introgression from close relatives (as in *Fundulus grandis*; Oziolor et al. 2019). Despite this limitation, the lack of parallelism at the SNP level provides some indication that introgression is unlikely to be the source of adaptive alleles. Dramatically greater overlap between the two Welsh comparisons (WWA and SWA) and a bias toward shared outlier SNPs rather than scaffolds, further supports the single origin of the Welsh mine populations and provides a clear contrast with the degree of outlier overlap with mine populations that evolved in other regions. It is possible that the difference in distribution of overlap between scaffolds and SNPs is due to a limited role of parallelism at the level of individual nucleotides, but greater convergence at the genic level (Conte et al. 2012). However, the sparse sampling inherent to the ddRAD approach may mean that the specific adaptive sites are not captured in the analysis (Lowry et al. 2017) and there may be more substantial sharing and parallelism of adaptive SNPs across independently derived mine populations.

A polygenic basis to differentiation in *S. uniflora* is at odds with previous investigations of heavy metal tolerance in *Silene.* Using controlled crosses and hydroponic experiments, these studies indicated that both zinc and copper tolerance have relatively simple genetic bases and are not controlled by the same molecular mechanisms (Schat et al. 1996; Schat and Vooijs 1997). The simple architecture for copper tolerance in *S. vulgaris* is also supported by the recent discovery of two related ATPase copper transporters which additively contribute to copper tolerance (Li et al. 2017). The potential for polygenic convergence in *S. uniflora* is further supported by gene ontology enrichment analysis of the subset of genes found on the 34 scaffolds which were outliers in all four pairwise comparisons. This group was significantly enriched for genes involved in metabolism of reactive oxygen species and the regulation of salicylic acid (supplementary table S6, Supplementary Material online), which are critical in responses to cold, salt, drought, and heavy metal stresses (Khan et al. 2015). Further systematic investigation of gene functions revealed that 15 genes have well-supported roles in processes that are relevant to differentiation between coastal and mine plants: eight associated with salt stress, eight with heavy metal stress and four with root development and morphology (supplementary table S7, Supplementary Material online). This points to a potential trade-off in the molecular processes which govern mine-coast differentiation, with selection against salt tolerance alleles in mines and against metal tolerance alleles in coastal environments. Alternatively, some alleles for genes that contribute to metal tolerance may be conditionally neutral in coastal plants and under positive selection in the mine environment. In this latter scenario, we might expect a higher incidence of metal tolerance among coastal population, but further work is needed to establish which model underlies local adaptation.

The exact mechanism of zinc tolerance in *Silene* is not well understood. However, hydroponic experiments with mine and coastal *S. uniflora* demonstrated that mine plants grown in zinc-contaminated media accumulate a higher proportion of absorbed zinc in the roots relative to their shoots whereas the reverse is true for coastal plants (Baker 1978). Additional research in *S. vulgaris* indicates that zinc uptake into tonoplast vesicles of zinc-tolerant *S. vulgaris* is higher than in nontolerant plants (Chardonnens et al. 1999). In our study, three genes on outlier scaffolds (*PSD2*, *WRKY23*, and *RWP1*) have direct links to these physiological differences between tolerant and nontolerant *Silene*: 1) *PSD2* encodes a form of phosphatidylserine decarboxylase which is located in the tonoplast (Nerlich et al. 2007), confers cadmium tolerance in *Saccharomyces cerevisiae* (Gulshan et al. 2009) and produces phosphatidylethanolamine, which is involved in zinc homeostasis in *Pseudomonas fluorescens* (Appanna et al. 1995); 2) *WRKY23* is a transcription factor that regulates root development by altering auxin distribution through the control of flavanol biosynthesis in *Arabidopsis thaliana—*overexpression of *WRKY23* increases quercetin root concentrations (Grunewald et al. 2012). Quercetin is a very efficient chelator of heavy metals (i.e., a molecule that binds metal ions) and supplementation of wild type *A. thaliana* with quercetin stimulates root growth in the presence of zinc ions (Keilig and Ludwig-Müller 2009); and 3) *RWP1* is required for the production of the cell wall polymer suberin. In *A. thaliana*, *RWP1* mutants lack suberin and have increased root permeability for NaCl (Gou et al. 2009). Furthermore, *Esb1* mutants have increased levels of root suberin, which both decreases accumulation of cadmium, manganese, and zinc in the shoots and increases accumulation of sodium in the shoots (Baxter et al. 2009).

Parallel evolution is expected to be facilitated in spatially structured environments when loci have large, spatially antagonistic fitness effects (Chevin et al. 2010). Evidence of such trade-offs in wild plants is lacking, with loci displaying conditional neutrality more commonly detected (Lowry et al. 2009; Hall et al. 2010; Anderson et al. 2011). The dual effect of high suberin levels on restriction of zinc ions to the roots and exposure of the shoots to sodium raises the possibility of a direct trade-off in suberin production and opens the possibility of antagonistic pleiotropy at *RWP1* influencing the parallel evolution of zinc-tolerant populations. Of the three genes, only the scaffold containing *RWP1* had consistently lower genetic diversity in the mine populations (paired *t*-test, two-sided, *P* = 0.030), whereas for *WRKY23* and *PSD2* diversity was only lower in the mines from West Wales and Ireland (supplementary table S4, Supplementary Material online). These findings further support the polygenic nature of parallel adaptation in *S. uniflora* and the potential importance of antagonistic pleiotropy in the rapid evolution of differentially adapted populations.

In a rapidly changing world, the adaptability of species will be critical for their long-term persistence. This study shows that some species will be capable of responding quickly to strong selection pressures across their range. We argue that plant species with sufficient genetic variation may adapt quickly to a single physiological stress repeatedly in different places, while using subtly different genetic mechanisms. As in *S. uniflora*, those species that evolved to survive in environments with natural sources of high abiotic stress, but which do not compete well in low-abiotic stress/high-biotic competition environments, may be particularly well suited to cope with the ongoing human modification of the planet. Alongside evidence of widespread local adaptation to different environmental conditions in other species (Fournier-Level et al. 2011; Papadopulos et al. 2014), our findings indicate that while it may be possible to predict which species will adapt to specific environments, the underlying genetic basis to that adaptation may be considerably more variable than is currently understood from the limited number of well-studied examples (Bay et al. 2018; Fitzpatrick et al. 2018; Oomen et al. 2020). In order to be accurate, predictions of evolutionary responses to environmental change from genomic data will need to account for the possibility that multiple genetic architectures can produce similar phenotypic responses.

## Materials and Methods

### Sample Collection

Four focal mine sites where *S. uniflora* was known to occur were selected for sampling; Grogwynion (West Wales; WWA; worked 1588–1889 C.E.; Hartley 2009), White Rock (Swansea, South Wales; SWA; 1736–1928; Hughes 2000), Priddy Pools (Somerset, South-West England; ENG; 1850–1908, evidence of Roman mining; Gough 1967) and Ross Island (Co. Kerry, South-West Ireland; IRE; 1707–1829, evidence of Bronze Age mining; O’Brien 2020). For three of these sites (WWA, ENG, and IRE), metal tolerance has previously been tested (Baker 1978; Schat et al. 1996). White Rock was also located near a previously tested population in Morriston, Swansea (Baker 1978) that no longer exists. The BSBI Database was used to identify the nearest accessible coastal populations to each mine. See supplementary table S8, Supplementary Material online for population coordinates. At each of the eight populations, leaf tissue was sampled from 30 individuals and preserved for DNA extraction in fine mesh silica gel. Individuals were sampled at least one m apart and samples were collected at even intervals across the extent of each population. At each site, we collected seeds from a minimum of 12 individuals, which were then dried and stored separately with silica gel. For assembly of a draft genome, cuttings from a single coastal individual were collected in Tresaith (West Wales), propagated and self-fertilized to produce an inbred F1 (SUTF1P) with reduced heterozygosity.

### Phenotyping

Root elongation experiments were conducted to determine the level of zinc and copper tolerance in each population (Baker 1978). Seeds were germinated in groups of eight (one seed per population) on ¼× Murashige-Skoog media in 1% agar with no supplemental heavy metals (control treatment), 24 µM copper sulfate (copper treatment) or 459 µM zinc sulfate (zinc treatment). Twenty graduated plates were prepared per treatment and the positions of populations within plates was determined using a random seed. Plates were placed upright in a germination cabinet with a 12-h light/dark cycle for 10 days and then photographed using a digital camera. Radicle length of all seedlings with emerged cotyledons was measured using ImageJ v1.8.0. Zinc and copper tolerance were calculated as the radicle length in the treatment divided by the mean length in the control for each population. Six individuals per population germinated on control media were grown into adults and zinc tolerance was assessed using deep water culture. To do this, cuttings from each individual were rooted in a mist propagator for 2 weeks before being transferred to a deep-water culture set up with 1/10× Hoagland’s solution. After acclimatization for 1 week, the plant roots were stained using a suspension of activated charcoal and rinsed with ddH_2_O, the solution was refreshed and 600 µM zinc sulfate was added. After a further 2 days root growth was inspected by eye—the presence of unstained root tips (i.e., ongoing root growth) was taken as confirmation of zinc tolerance (Schat et al. 1996; Bratteler et al. 2006a).

### Genome Assembly

DNA was extracted from silica dried leaf tissue using Qiagen DNeasy Plant tissue kits. DNA quality was assessed using agarose gel electrophoresis and DNA was quantified using a Promega Quantus fluorometer with Quantifluor dsDNA kits. For draft genome assembly, four NEBnext Ultra II libraries were prepared for SUTF1P and each was sequenced using illumina MiSeq v3 600 bp PE cartridges. Adapter and quality trimming were performed using cutadapt v2.1 (Martin 2011) and Trimmomatic v0.36 (Bolger et al. 2014) (minimum quality = 15, minimum length = 64). Overlapping read pairs were merged using Abyss-mergepairs (Jackman et al. 2017) and nonoverlapping pairs merged using konnector v2.0 (Vandervalk et al. 2015) with a bloom filter containing merged and unmerged reads for all libraries (kmer length = 96, bloom filter FPR = 1.01%). illumina reads were assembled into contigs using Abyss v2.0 (Jackman et al. 2017) with a kmer length = 241—selected after estimation with kmergenie v1.7048 and Abyss runs with kmers = 96/127/151. To scaffold the assembly, the same individual was sequenced using an Oxford Nanopore MinION (Three R9 flow cells and one R9.4 flow cell with SQK-NSK007 kits). Nanopore reads were corrected with Proovread v2.12 (Hackl et al. 2014) using the processed illumina reads. Redundans v0.14a (Pryszcz and Gabaldón 2016) was used to reduce contig redundancy caused by heterozygosity (minimum identity 95%) and scaffold contigs using the corrected nanopore data. Abyss-sealer (Paulino et al. 2015) was used to fill gaps in the scaffolded assembly (kmers = 94/89/84) and completeness was assessed with BUSCO v3 (Benchmarking Universal Single-Copy Orthologs; complete and fragmented = 78.5%, supplementary table S5, Supplementary Material online). Augustus (Stanke et al. 2006) was used to predict genes in the genomic scaffolds using the annotation training files from *Solanum lycopersicum*. The resulting predicted amino acid sequences were BLASTp-searched (Camacho et al. 2009) against the *Arabidopsis thaliana* proteome (Araport11) and only the best scoring hit from each predicted amino acid sequence was retained.

### Genotyping

Double-digest RAD sequencing was performed following a modified protocol of Peterson et al (2012) detailed in Papadopulos et al (2019) and restriction was performed using *Eco*RI-HF and *Msp*I. For this study, size selection was conducted with a pippin prep (468–546 bp) and one pool of 230 uniquely barcoded individuals was sequenced on five lanes of an illumina HiSeq 2500 (100 bp, PE) at the Earlham Institute. Raw reads were demultiplexed, trimmed to 90 bp and low-quality reads were discarded, resulting in an average of 4.76 M reads per sample (s.d. 2.01 M). Reads were mapped to the draft genome using bowtie v2.3.4 (Langmead and Salzberg 2012) in end-to-end mode and excluding reads with low mapping quality (Q < 20). SNPS were called from the resulting BAM files using gstacks v2.0b (Rochette et al. 2019), 14 samples were excluded from further analysis due to low coverage. Genotypes for SNPS with less than 20% missing data were extracted in VCF and RADpainter format using Populations v2.0b (Rochette et al. 2019). In total, 216 individuals were genotyped at 74,064 SNPs.

### Evolutionary Genetics

Population genetic structure across *S. uniflora* was assessed using PCA implemented in adegenet v2.1.3 (Jombart 2008) in R and genetic coancestry was estimated using the haplotype-based inference method of fineRADstructure v0.3.2 (Malinsky et al. 2018). AMOVA was conducted in Arlequin v3.5.2.2 (Excoffier and Lischer 2010) To assess patterns of isolation by distance, pairwise genetic differentiation between the sampled populations (Weir and Cockerham’s *F*_ST_) was calculated using Arlequin v3.5.2.2 (Excoffier and Lischer 2010), pairwise geographic distances between populations were calculated with the distm function in the geosphere R package and isolation by distance estimated in R using linear regression. Tajima’s *D* was calculated for 20 kb sliding windows in VCFtools v0.1.16 (Danecek et al. 2011) and averaged over the subset of windows for which *D* could be calculated in all populations. To identify the isolation by distance signature expected from parallel versus single origins of the mine populations, we conducted simulations in SLiM v3.3.2 under two scenarios: independent colonization of mines from the nearest coastal population and non-independent colonization of mines from the same individual coastal population. In the latter case, the “founding” coastal population was randomly chosen in each independent iteration of the simulation. All simulations were initiated with a burn-in period of 100,000 generations and a population size of 10,000 individuals. Each individual in the population was diploid and hermaphroditic, and generations were nonoverlapping (i.e., Wright-Fisher simulations). To track genetic relationships among populations, we simulated a single chromosome that was 50,000 bp long with a uniform mutation rate of 7.5 × 10^−9^—based on estimates for *S. latifolia* (Krasovec et al. 2018)—and a recombination rate of 4.0 × 10^−9^—based on the genetic map length (446 cM; Bratteler et al. 2006b) and genome size (1.13 Gb) of *S. vulgaris* (Pellicer and Leitch 2020). In the 100,000th generation, two populations (p1 and p2) were colonized with 500 individuals each from the ancestral population. These two populations represented those that initially colonized Ireland and the west coast of England/Wales at the end of the Last Glacial Maximum. Subsequent stepwise colonization of populations (i.e., p2 -> p3 -> p4), representing coastal populations, occurred every 20 generations until there were four coastal populations in the 100,040th generation. Coastal populations were always founded with 500 individuals and population sizes increased to 1,000 individuals ten generations after a population was initially founded. After colonization, p1 and p2 exchanged migrants at a rate of 0.00001 per generation, p2 and p3 at a rate of 0.0001, and p3 and p4 at a rate of 0.0001. P1 through p4 were therefore effectively arranged along a line and migration rates between nonadjacent populations were equivalent to the product of migration rates connecting them. Ten thousand generations after the coastal populations were founded, 100 individuals were used to found each of four populations meant to reflect those found in mine environments. After founding the mine populations, these populations exchanged migrants with the nearest coastal population at a rate of 0.0002. All populations then evolved for an additional 100 generations. At the end of the simulations (i.e., at generation 1,10,150), we calculated and output *F*_ST_ between each of the four coastal populations (all pairwise comparisons) and each of the four mine populations. We ran 100 independent replicates for each of the three colonization scenarios described above.

To further establish the evolutionary relationships between the populations, the data set was pruned to 7,037 SNPs using a linkage disequilibrium threshold of 0.1 and minor allele frequency threshold of 0.05, and the phylogenetic tree estimated with 1,000 bootstrap replicates using the maximum-likelihood approach implemented in SNPhylo v2 (Lee et al. 2014). This reduced data set was then used to explore the possibility of migration and introgression between the populations using Treemix v0.1.15 (Pickrell and Pritchard 2012). For the maximum-likelihood estimation of the tree in Treemix, one to ten migration edges were fitted and the number of edges that explained 99.8% of the variance selected as the best model. Using the fourpop function in Treemix, *f*4 statistics (Reich et al. 2009) were calculated for all population quartets to assess whether relationships between the populations deviated significantly (after Dunn–Bonferroni correction) from tree likeness. The premise of the *f*4 statistic and our test is that for any four populations there are three possible trees [((A, B),(C, D)); ((A, C),(B, D)); and ((A, D),(B, C))]. If ((A, B),(C, D)) is the correct tree, the allele frequency difference between A and B will not be correlated with the frequency difference between C and D, that is, the correlation in frequency differences (*f*4) would not deviate from zero (Reich et al. 2009). For each quartet of populations in our sample, we determined the correct tree based on figure 3*A* and tested whether *f*4 significantly deviated from zero using the *z*-score.

To investigate the level of parallel evolution at the molecular level, we calculated Weir and Cockerham’s *F*_ST_ at all variable sites in pairwise comparisons between the geographically proximate mine-coast pairs using VCFtools v0.1.16. SNPs falling in the upper 95% percentile of values in each pairwise comparison were designated as outlier loci and scaffolds containing one of more outlier SNPS were designated as outlier scaffolds. Overlap of outlier SNPs and scaffolds was visualized using upsetR v1.4.0 (Conway et al. 2017) and significance of overlap was assessed using SuperExactTest v1.0.7 (Wang et al. 2015). To investigate the possible functions of genes in outlier regions, all genes on the outlier scaffolds that were in common across the four pairwise mine-coast comparisons were subjected to gene ontology enrichment analysis performed in topGo v3.11 (Alexa and Rahnenfuhrer 2020) using the “elim” algorithm and Fisher’s Exact tests to assess significance. Further assessments of gene functions were made from The *Arabidopsis* Information Resource (TAIR) descriptions and associated references. Systematic searches were performed using gene names with and without the terms “stress” and “heavy metal” using Google Scholar.

## Supplementary Material

Supplementary data are available at *Molecular Biology and Evolution* online.

## Supplementary Material

msab141_Supplementary_Data
