# Supplementary material for: Rapid Parallel Adaptation to Anthropogenic Heavy Metal Pollution
Source: Mol Biol Evol. 2021 May 5;38(9):3724–36. doi: 10.1093/molbev/msab141 (PMC8382892; doi:10.1093/molbev/msab141)
Supplement: msab141_Supplementary_Data [file msab141_supplementary_data.zip › Silene_Parallel_SI_MBE_revision.pdf]

Supplementary Information for:

Rapid Parallel Adaptation to Anthropogenic Heavy Metal Pollution

Alexander S.T. Papadopulos[1,2]*, Andrew J. Helmstetter[2,3], Owen G. Osborne[1], Aaron A. Comeault[1], Daniel P. Wood[1], Edward A. Straw[2,4], Laurence Mason[2], Michael F. Fay[2,5], Joe Parker[2,6], Luke T. Dunning[7], Andrew D. Foote[1,8], Rhian J. Smith[2] & Jackie Lighten[9]

[1]Molecular Ecology and Fisheries Genetics Laboratory, Environment Centre Wales, School of Natural Sciences, Bangor University, Bangor, LL57 2UW, UK

[2]Royal Botanic Gardens, Kew, Richmond, Surrey TW9 3AB, UK

[3]Institut de Recherche pour le Développement (IRD), UMR-DIADE, 911 Avenue Agropolis, BP 64501, 34394 Montpellier, France

[4]Centre for Ecology, Evolution & Behaviour, Department of Biological Sciences, School for Life Sciences and the Environment, Royal Holloway University of London, Egham, Surrey, TW20 0EX, UK

[5]School of Plant Biology, University of Western Australia, Crawley, WA 6009, Australia

[6]National Biofilms Innovation Centre, Department of Biological Sciences, University of Southampton, SO14 1BJ, UK.

[7]Department of Animal and Plant Sciences, University of Sheffield, Sheffield, UK

[8]Department of Natural History, Norwegian University of Science and Technology, Trondheim, Norway

[9]Biosciences, University of Exeter, Stocker Road, Exeter EX4 4PY, UK

*Correspondence to: a.papadopulos@bangor.ac.uk

Figure S1. Co-ancestry matrix from finRADstructure clustered by population.

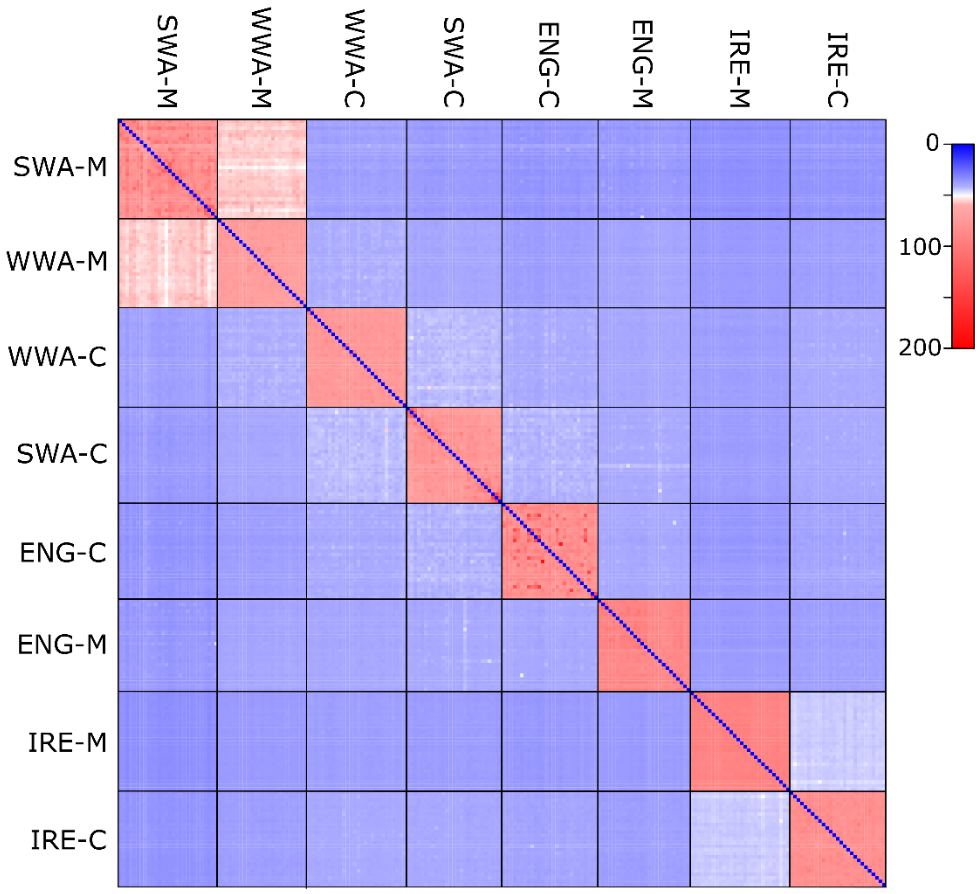

Figure S2. Tukey's test of association between number of outlier SNPs and scaffold length. 95% family-wise confidence levels

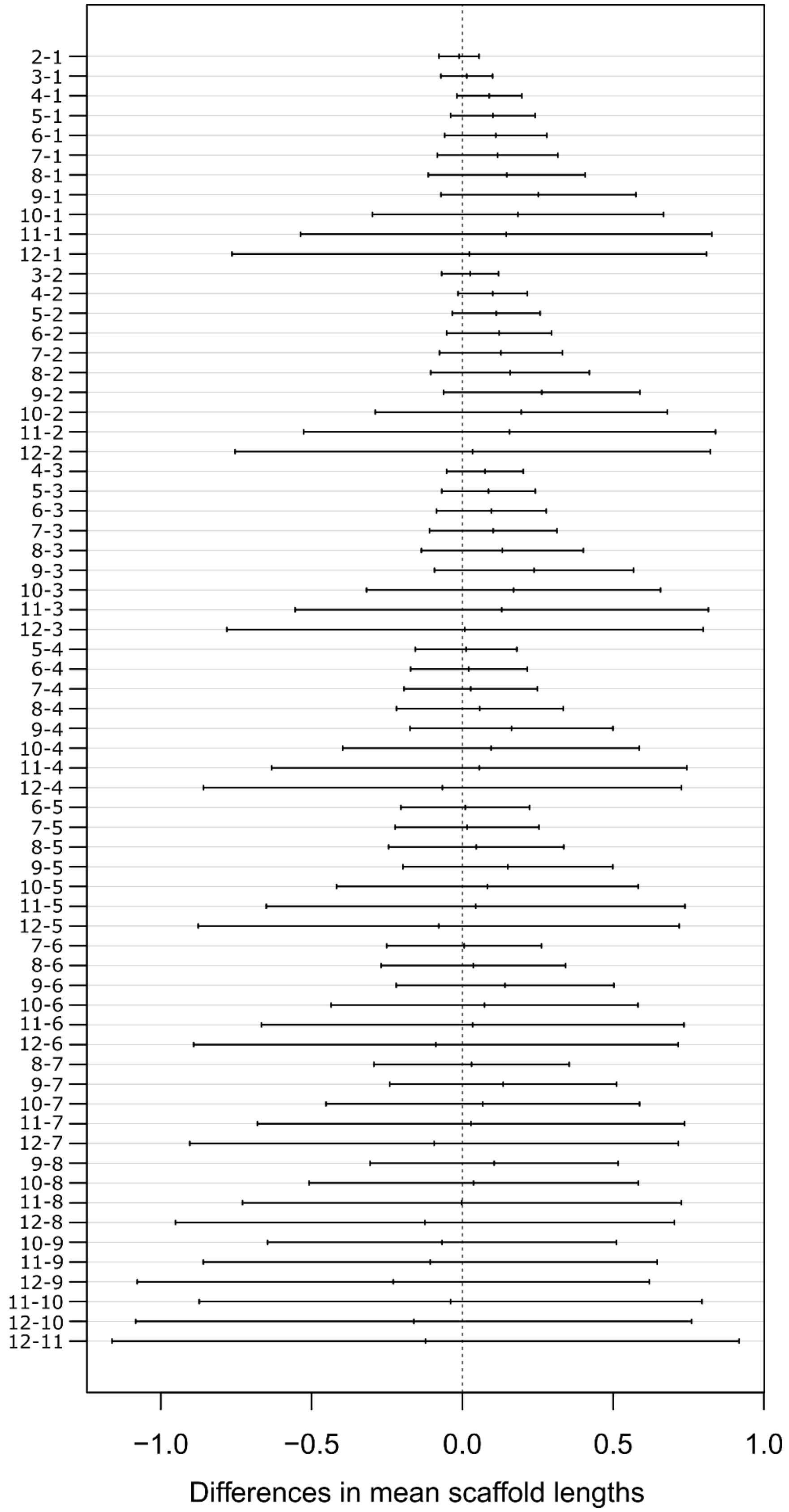

Table S1. Heavy metal concentration recorded at mines included in this study

Mine	Zinc (ppm)	method	Copper (ppm)	Cu method	Lead (ppm)	Pb method
WWA	2410[a]	AAS	180[d]	AAS	10k-52k[f]	ICP-AES
SWA	14490[b]	XRF	11350[b]	XRF	14000[b]	XRF
ENG	17000[a]	AAS	35[e]	AAS	21038[e]	AAS
IRE	48075[c]	XRF	12069[c]	XRF	52633[c]	XRF

Measurement method: **AAS** - Atomic Absorption Spectrophotometry; **XRF** - X-Ray Fluorescence; ICP-**AES** - Inductively Coupled Plasma Atomic Emission Spectroscopy. **References:** [a]Baker AJM. 1978. Ecophysiological aspects of zinc tolerance in Silene maritima With. New Phytol. 80:635–642; [b]Burka G. 2017. Personal communication; [c]Chouinard C. 2017. Elemental contamination of an ancient copper mine in Killarney, Ireland. Thesis; [d]Baker AJM. 1974. Heavy metal tolerance and population differentiation in Silene maritima with. PhD; [e]Allison S. 2002. Distribution and biogeochemical cycling of trace metals in soils at an abandoned lead mining and smelting. PhD; [f]Palumbo-Roe B, Klinck B, Banks V, Quigley S. 2009. Prediction of the long-term performance of abandoned lead zinc mine tailings in a Welsh catchment. J. Geochemical Explor. 100:169–181.

Table S2. Pairwise genetic differentiation (F_{ST}) between populations

	**WWA-M**	**ENG-M**	**IRE-M**	**SWA-M**	**WWA-C**	**ENG-C**	**IRE-C**
**ENG-M**	0.427	-	-	-	-	-	-
**IRE-M**	0.552	0.578	-	-	-	-	-
**SWA-M**	0.182	0.427	0.540	-	-	-	-
**WWA-C**	0.295	0.336	0.464	0.314	-	-	-
**ENG-C**	0.341	0.349	0.473	0.340	0.244	-	-
**IRE-C**	0.365	0.389	0.430	0.365	0.269	0.287	-
**SWA-C**	0.295	0.309	0.440	0.307	0.199	0.219	0.253

Table S3. Hierarchical populations structure using Analyses of Molecular Variance (AMOVA)

Grouped by	Source of variation	Nested within	% variation
Habitat (Mine/Coast)	Among Habitat		1.54
	Among Populations	Habitat	33.71
	Among Individuals	Populations	10.30
	Within Individuals		54.45
Region (SWA/WWA/ENG/IRE)	Among Region		2.09
	Among Populations	Region	32.92
	Among Individuals	Populations	10.34
	Within Individuals		54.65

Table S4. Genetic diversity and Tajima's D for each population

**Population**	**Tajima's D**	**π**	**π *WRKY23***	**π *RWP1***	**π *PSD2***
WWA-M	0.314	0.049	0.011	0.056	0.105
WWA-C	0.207	0.064	0.088	0.188	0.137
SWA-M	0.013	0.056	0.087	0.075	0.130
SWA-C	0.160	0.071	0.039	0.101	0.091
ENG-M	0.401	0.044	0.088	0.105	0.066
ENG-C	0.203	0.065	0.079	0.194	0.054
IRE-M	0.336	0.028	0.003	0.000	0.018
IRE-C	0.280	0.059	0.108	0.099	0.046

Table S5. Genome assembly statistics

**Assembly statistics**	**bp**
n scaffolds	269915
n scaffolds > 5kb	36751
Largest contig	151586
Total length	769407286
GC (%)	36.88
N50	4660
N75	2183
L50	40594
L75	101673
N's per 100kbp	982.85

**BUSCOs**	**n (%)**
Complete BUSCOs	1501 (64.5)
Complete and single-copy BUSCOs	1368 (58.8)
Complete and duplicated BUSCOs	133 (5.7)
Fragmented BUSCOs	326 (14.0)
Missing BUSCOs	499 (21.5)

Table S6 Gene ontology enrichment analysis for the 34 outlier scaffolds which were common to all ecotype pairs.

GO ID	Term	Annotated	Significant	Expected	p-value
GO:0042938	Dipeptide transport	64	3	0.08	0.000058
GO:0010337	Regulation of salicylic acid metabolic process	37	2	0.04	0.00087
GO:0042939	Tripeptide transport	43	2	0.05	0.00117
GO:0010310	Regulation of hydrogen peroxide metabolic process	53	2	0.06	0.00178
GO:0000904	Cell morphogenesis involved in differentiation	634	4	0.75	0.00612
GO:0051704	Multi-organism process	4313	11	5.09	0.00663
GO:0009631	Cold acclimation	114	2	0.13	0.00794
GO:0033036	Macromolecule localization	775	4	0.91	0.01224
GO:0009860	Pollen tube growth	413	3	0.49	0.01233
GO:0009617	Response to bacterium	1793	6	2.11	0.01603
GO:0009932	Cell tip growth	472	3	0.56	0.01759
GO:0048868	Pollen tube development	548	3	0.65	0.02598
GO:0008104	Protein localization	552	3	0.65	0.02647
GO:0048588	Developmental cell growth	569	3	0.67	0.02862
GO:0051707	Response to other organism	3274	8	3.86	0.03032
GO:0043207	Response to external biotic stimulus	3274	8	3.86	0.03032
GO:0070727	Cellular macromolecule localization	582	3	0.69	0.03032
GO:0009607	Response to biotic stimulus	3275	8	3.86	0.03037
GO:0044706	Multi-multicellular organism process	710	3	0.84	0.04992
GO:0009856	Pollination	710	3	0.84	0.04992

Table S7. Functional information for genes on 34 common outlier scaffolds

Scaffold	Gene ID	Locus Identifier	Gene Symbol	Relevant function	Evidence	References
scaffold14499	g46038	AT2G02450	LONG VEGETATIVE PHASE 1 (LOV1)	Cold	Regulates the plant cold response by positive regulation of the cold response genes COR15A and KIN1. Interacts with CBF/DREB1 genes (CBF2)	https://doi.org/10.1371/journal.pone.0000642
scaffold00101	g707	AT4G25470	C-REPEAT/DRE BINDING FACTOR 2 (CBF2)	Cold; Drought; Salt	Key role in cold tolerance and plays role in drought and salt tolerance. Also induced under cadmium stress in rice	https://doi.org/10.1093/jxb/err279 https://doi.org/10.1007/s11104-009-0116-9 https://doi.org/10.1093/jxb/erp300
scaffold00735	g4576	AT5G17310	UDP-GLUCOSE PYROPHOSPHORYLASE 2 (UGP2)	Cold; Roots	Affects root length expression changes under cold stress and heat shock in *A. thaliana*	https://doi.org/10.1093/pcp/pcp052
scaffold12242	g41152	AT1G32060	PHOSPHORIBULOKINASE (PRK)	Cold; Salt	Component of Calvin cycle. Expression inhibited by cold and salt stress in *Mesembryanthemum crystallinum.*	https://doi.org/10.1007/BF00028789
scaffold17088	g51384	AT5G55190	RAN GTPASE 3 (RAN3)	Cold; Salt	Expression increases in response to cold, salt and ABA stress, and *A. thaliana* mutants are sensitive to salt and ABA treatment. CBF2 expression levels were lower in a atran1/atran3 double mutant 10.1371/journal.pone.0154787	https://doi.org/10.1371/journal.pone.0154787
scaffold04408	g19664	AT5G54270	LIGHT-HARVESTING CHLOROPHYLL B-BINDING PROTEIN 3 (LHCB3)	Drought; Inhibited by Zinc	Involved in stomatal response to abscisic acid and therefore important for drought stress response Zinc inhibits Photosystem II core complex.	https://doi.org/10.3390/ijms19030787 https://doi.org/10.1104/pp.66.6.1174 https://doi.org/10.1007/BF00042010
scaffold09338	g34010	AT3G54140	ARABIDOPSIS THALIANA PEPTIDE TRANSPORTER 1 (AtPTR1)	Heavy metal	Possible role in glutathione transport and cadmium tolerance.	https://doi.org/10.1016/j.febslet.2007.04.047
scaffold00902	g5465	AT3G53000	PHLOEM PROTEIN 2-A15 (PP2-A15)	Heavy metal	Protein specifically expressed in *Arabis paniculata* (hyper accumulator) in response to zinc stress.	https://doi.org/10.1016/j.chemosphere.2010.10.030
scaffold00735	g4578	AT5G57190	PHOSPHATIDYLSERINE DECARBOXYLASE 2 (PSD2)	Heavy metal	Located in tonoplast and produces phosphatidylethanolamine. In *S. cervisiae* is required for cadmium tolerance. Pseudomonas fluorescens produces phosphatidylethanolamine to avoid effects millimolar amounts of metals including Zinc	https://doi.org/10.1091/mbc.e09-06-0519 https://doi.org/10.1111/j.1574-6968.1995.tb07753.x
scaffold06199	g25472	AT2G47260	WRKY DNA-BINDING PROTEIN 23 (WRKY23)	Heavy metal; Roots	WRKY23 regulates root development by affecting auxin distribution through the control of flavonol biosynthesis in *A. thaliana*. It increases quercetin root concentrations in overexpression mutants, whereas WRKY23 reduction of expression roots lack quercetin-rhamnoside-glucoside. Quecetin chelates metal ions can induce root growth in plants inhibited by Zinc.	https://doi.org/10.1073/pnas.1121134109
scaffold00074	g528	AT4G29010	ABNORMAL INFLORESCENCE MERISTEM (AIM1)	Heavy Metal; Salt	Key component of jasmonic acid (JA) synthesis and benzoic acid (BA) syntheses. JA is involved in both salt stress responses and heavy metal responses in plants. BA is known to be important for salt stress.	https://doi.org/10.1093/jxb/erw202 https://doi.org/10.1631/jzus.B1700191
scaffold01631	g8909	AT5G33340	CONSTITUTIVE DISEASE RESISTANCE 1 (CDR1)	Heavy Metal; Salt	Triggers expression of PR1 and PR2 which are part of salicylic acid pathway. Overexpression of PR1 and PR3 in tobacco plants leads enhanced tolerance to salt and heavy metals	https://doi.org/10.1038/sj.emboj.7600086. https://doi.org/10.1016/j.micres.2018.04.008 https://doi.org/10.1016/j.micres.2018.04.008

Table S7. Continued…

Scaffold	Gene ID	Locus Identifier	Gene Symbol	Relevant function	Evidence	References
scaffold02487	g12589	AT5G41040	REDUCED LEVELS OF WALL-BOUND PHENOLICS 1 (RWP1)	Heavy Metal; Salt	Required for suberin synthesis, lack of suberin in RWP1 mutants in *A. thaliana* leads to increased salt sensitivity. Suberin also contributes to blocking metal uptake from roots to shoots.	https://doi.org/10.1073/pnas.0905555106 https://doi.org/10.1371/journal.pgen.1000492
scaffold06199	g25474	AT2G17030	SKP1/ASK-INTERACTING PROTEIN 23 (SKIP23)	Heavy Metal; Salt	Differentially expressed under salt and mercury treatment in *Medicago trunculata*	https://doi.org/10.1007/s10142-015-0438-z
scaffold00284	g1919	AT3G57240	BETA-1,3-GLUCANASE 3 (BG3)	Pathogen	Response to fungal pathogens	https://doi.org/10.1093/molbev/msm024
scaffold06199	g25471	AT2G03200	ATYPICAL ASPARTIC PROTEASE IN ROOTS 1 (ASPR1)	Roots	Involved in regulation of root development in *A. thaliana*. Overexpression leads to shorter primary roots and fewer lateral roots. Possible role in redox signalling in roots	https://doi.org/10.1093/jxb/erz059
scaffold00463	g3016	AT2G31280	CONSERVED PEPTIDE UPSTREAM OPEN READING FRAME 7 (CPuORF7)	Roots	Loss of function and overexpression mutants in *A. thanliana* show that LHL3 plays a positive role in xylem differentiation downstream of auxin	https://doi.org/10.1093/pcp/pct013
scaffold00556	g3575	AT5G50010	Basic helix-loop-helix 145 (SACL2)	Salt	Possible role in salt tolerance - quadruple T-DNA insertion mutant of the SAC51 family showed increased salt sensitivity in *A. thaliana*	https://doi.org/10.1111/tpj.14448
scaffold00101	g715	AT4G23160	CYSTEINE-RICH RLK (RECEPTOR-LIKE PROTEIN KINASE) 8 (CRK8)	Salt	Salt stress response. Germination of Crk8 knockout in *A. thaliana* is inhibited by NaCl	https://doi.org/10.1371/journal.pgen.1005373
scaffold00902	g5467	AT3G53030	SER/ARG-RICH PROTEIN KINASE 4 (SRPK4)	Salt	Phosphorylates SCL30 and RSp31. RSp31 undergoes increased splicing under salt stress. SCL30 constitutively phosphorylated by SRPK4.	https://doi.org/10.1186/1471-2164-15-431
scaffold04926	g21384	AT3G54420	HOMOLOG OF CARROT EP3-3 CHITINASE (EP3)	Wound	Highly expressed in roots. Response to wounding, but no increased expression under abiotic stress.	https://doi.org/10.1271/bbb.80837
scaffold43030	g91266	AT1G23360	2-phytyl-1,4-naphthoquinone methyltransferase (MENG)	-	-	-
scaffold03268	g15586	AT3G19910	RING/U-box superfamily protein (CTL18)	-	-	-
scaffold14742	g46598	AT1G01900	Subtilisin-like serine protease (SBTI1.1)	-	-	-
scaffold02487	g12592	AT3G01990	ACT DOMAIN REPEAT 6 (ACR6)	-	-	-
scaffold09338	g34011	AT5G01180	ARABIDOPSIS THALIANA PEPTIDE TRANSPORTER 1 (AtPTR5)	-	-	-
scaffold00284	g1918	AT3G57270	BETA-1,3-GLUCANASE 1 (BG1)	-	-	-
scaffold28068	g70516	AT5G23530	CARBOXYESTERASE 18 (CXE18)	-	-	-
scaffold43030	g91265	AT1G23380	KNOTTED1-LIKE HOMEOBOX GENE 6 (KNAT6)	-	-	-
scaffold04408	g19665	AT5G54280	MYOSIN 2 (ATM2)	-	-	-
scaffold02103	g11039	AT3G07680	P24 SUBFAMILY BETA 2 (P24BETA2)	-	-	-
scaffold00735	g4577	AT1G03050	PHOSPHATIDYLINOSITOL BINDING CLATHRIN ASSEMBLY PROTEIN 5A (PICALM5A)	-	-	-
scaffold18761	g54571	AT3G18680	PLASTID 55 UMP KINASE (PUMPKIN)	-	-	-
scaffold00284	g1916	ATCG00190	RNA POLYMERASE SUBUNIT BETA (RPOB)	-	-	-
scaffold00074	g527	AT2G27520	-	-	-	-
scaffold00074	g529	AT4G29000	-	-	-	-
scaffold00074	g530	AT4G28990	-	-	-	-
scaffold00101	g713	AT5G23400	-	-	-	-
scaffold00101	g714	AT1G21280	-	-	-	-
scaffold00101	g718	ATMG00710	-	-	-	-
scaffold00101	g720	AT4G29090	-	-	-	-
scaffold00284	g1917	AT1G74630	-	-	-	-
scaffold00284	g1921	AT5G07400	-	-	-	-
scaffold00284	g1923	AT4G00980	-	-	-	-

Table S7. Continued…

Scaffold	Gene ID	Locus Identifier	Gene Symbol	Relevant function	Evidence	References
scaffold00383	g2509	AT3G01410	-	-	-	-
scaffold00383	g2516	ATMG00860	-	-	-	-
scaffold00383	g2520	AT4G13320	-	-	-	-
scaffold00463	g3015	AT5G45190	-	-	-	-
scaffold00463	g3017	AT1G06640	-	-	-	-
scaffold00556	g3570	AT1G20400	-	-	-	-
scaffold00556	g3576	AT5G51560	-	-	-	-
scaffold00735	g4575	AT5G14720	-	-	-	-
scaffold00902	g5466	AT2G36630	-	-	-	-
scaffold01259	g7215	AT3G19184	-	-	-	-
scaffold01631	g8908	AT2G07760	-	-	-	-
scaffold01631	g8910	AT1G64830	-	-	-	-
scaffold01631	g8914	AT5G09430	-	-	-	-
scaffold02487	g12590	AT5G14080	-	-	-	-
scaffold03268	g15587	AT5G08310	-	-	-	-
scaffold04926	g21383	AT2G05642	-	-	-	-
scaffold06199	g25466	AT5G17410	-	-	-	-
scaffold06911	g27490	AT1G33710	-	-	-	-
scaffold06911	g27491	AT1G16220	-	-	-	-
scaffold09359	g34066	AT5G16210	-	-	-	-
scaffold12242	g41153	AT5G45590	-	-	-	-
scaffold28068	g70515	AT5G05180	-	-	-	-
scaffold119902	g154220	AT5G19890	-	-	-	-
scaffold168321	g177089	AT5G56190	-	-	-	-

Table S8. Population GPS coordinates

	Latitude	Longitude
WWA-M	52.331608	-3.887207
WWA-C	52.394825	-4.093914
SWA-M	51.635815	-3.933929
SWA-C	51.566044	-4.003153
ENG-M	51.256935	-2.650950
ENG-C	51.323284	-3.016996
IRE-M	52.034954	-9.539131
IRE-C	52.128418	-9.898941
